# A diffeomorphic aging model for adult human brain from cross-sectional data

**DOI:** 10.1038/s41598-022-16531-6

**Published:** 2022-07-25

**Authors:** Alphin J. Thottupattu, Jayanthi Sivaswamy, Venkateswaran P. Krishnan

**Affiliations:** 1 International Institute of Information Technology, Hyderabad, 500032 India; 2 TIFR Centre for Applicable Mathematics, Bangalore, 560065 India

**Keywords:** Computational biology and bioinformatics, Neuroscience

## Abstract

Normative aging trends of the brain can serve as an important reference in the assessment of neurological structural disorders. Such models are typically developed from longitudinal brain image data—follow-up data of the same subject over different time points. In practice, obtaining such longitudinal data is difficult. We propose a method to develop an aging model for a given population, in the absence of longitudinal data, by using images from different subjects at different time points, the so-called cross-sectional data. We define an aging model as a diffeomorphic deformation on a structural template derived from the data and propose a method that develops topology preserving aging model close to natural aging. The proposed model is successfully validated on two public cross-sectional datasets which provide templates constructed from different sets of subjects at different age points.

## Introduction

Human brain morphometry varies with respect to age, gender, and population. Since the human brain changes structurally with age, understanding the normative aging process from structural and functional images has been of interest both in general and within a specific population. Studies aimed at arriving at such an understanding, either use a longitudinal or a cross-sectional design^1^ for collecting images of the study cohort. The former is usually difficult as it is challenging to access a fixed cohort over an extended number of years and scan them repeatedly. A more pragmatic approach is based on a cross-sectional design where a set of individuals in different age range forms the cohort. This approach makes it easier to collect scans but their analysis requires a disentangling of the inter-subject variations from age-related changes which is not straight forward. A more elaborate treatment of the differences in such approaches can be found in Ref.^2^. Regardless of the data collection strategy, in computational anatomy, aging is typically modelled as a *continuous* deformation of a template image over time^3^. This modelling helps to derive any age-specific template from the model, develop subject specific growth trajectory and derive direct interpretations from the deformation field about the aging pattern. In this paper, we propose a method to develop such an aging model for healthy, adult human brain using cross-sectional data drawn from a population.

Since aging trend varies across individuals, longitudinal data-based age modelling typically starts with follow-up scans of a cohort. The continuous aging deformation for each individual can be accurately extracted by regression approaches^4–7^ as they find the best fit model for the data. The subject-specific trajectories are then mapped to a common space^8–12^ to define an aging model. The variability in inter-subject aging trends are also of interest and hence have been studied in longitudinal settings. A group-wise analysis of multiple elderly subjects in different age groups was done to understand aging in healthy adults versus Alzheimer’s disease using follow up scans of subjects collected over a short time span of up to 6.9 years^12^. Inter-subject variations have also been studied by considering a tubular neighbourhood for the deformation^13^. The spatio-temporal model^14^ also considers similar variations due to diseased data points in the dataset and uses partial least squares regression to compute normal aging deformations; this gives modes of aging and corresponding scores for each subject. Modelling of early brain development from longitudinal and cross-sectional image data, have used both parametric and non-parametric regression approaches^15–18^. Given the large volume change during this stage, these models in general relax the volume preserving deformation/diffeomorphism criteria in the model. Changes in the human brain at an early developmental stage is distinct from those due to aging of the adult brain as the latter has a more complex anatomy and the changes are much slower and more consistent over the entire age range^19,20^. Furthermore, since brain development is highly influenced by nature and nurture of an individual, a mature/adult brain shows complex variations across anatomy and smoother variations with aging across individuals^21–23^; such variations are termed as cross-sectional variations.

A cross-sectional design to perform a cohort based aging study allows creation of larger data sets compared to that with longitudinal data. In aging studies with a cross-sectional design, the inter-subject variability within an age group and across age groups is disentangled to some extent by developing templates which represent subjects in some small age interval^24–26^. Several such template data are publicly accessible even though the image-sets used for template generation are not publicly available^24,26,27^.

An early approach to develop an aging model from cross-sectional data for the adult brain employed a parametric regression approach based on weighted averaging^28^. More recently, a global template was created by co-registering given templates and a mapping between each of the given templates to this global template was defined as the aging model^29^. There is no attempt in this work to define a relation between the age points which implies the derived aging model is discrete. These methods are a departure from the notion of aging as a continuous deformation process acting on a template^3^.

We argue that an aging model developed by smoothly deforming a global template is more natural and attractive than one defined as a weighted average of image points in neighbourhoods^28^ or a discrete model^29^. Hence, given cross-sectional data in the form of templates, we explore the use of a diffeomorphic deformation of a global template as an aging model. The contributions of this paper are: a method to derive (i) a continuous aging model for a normal, adult brain from cross-sectional data covering a long age span and (ii) an aging model based on a diffeomorphic deformation of a global template that is more versatile than existing models.

## Methods

The proposed method derives an aging model from cross-sectional data of a cohort. We aim to address the case where the cross-sectional data is in the form of templates for different age groups as scans of subjects in the cohort are usually more difficult to source. It should be pointed that for a good representation of an age point, the number of subject scans used to construct the template has to be high since a small number of subjects will result in poor normalization^30^.

### Rationale

Aging of a mature, human brain is characterised by a deformation trend that is slow, spatially smooth and monotonic since atrophy, which is the hallmark of aging^22^, is reported to be slow and non-uniform^20,23,31,32^; the rate of atrophy being higher in the elderly age group compared to the younger age group. Hence, aging has to be modelled as a temporally non-uniform deformation. Regression approaches help to find the best fit model for such time series data by optimizing an initial image and path. Experiments with cross-sectional data have shown the regression results to be sensitive to the choice of initial image^7^. In the current problem of interest, due to the complex inter-subject anatomy variations, a joint optimization of the anatomy and path will therefore, result in an initial anatomy-dependent, best fit model whereas what is desired is a representation for aging (in a global space). Hence, a direct regression is inappropriate for age modelling from cross-sectional data.

**Figure 1:**
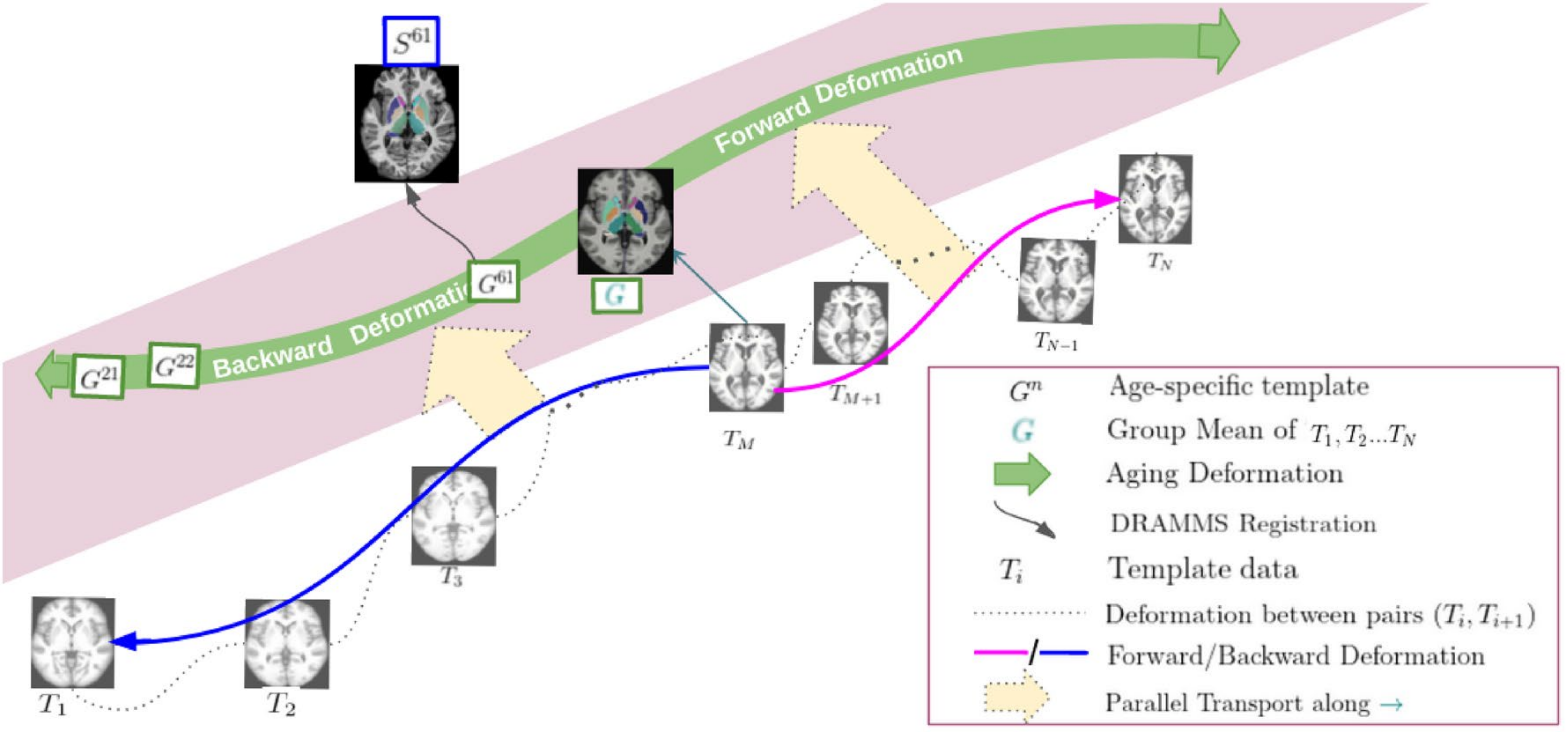
A schematic representation of the aging model. Included is an illustration of how the model maps a subject scan at age = 61 years to the global space.

The proposed model defines the aging process as a continuous deformation of a global template that represents the average anatomy represented by all the templates in the cross-sectional data. The model uses age-specific templates as the starting point and extracts the aging deformation across them. Separating non-aging variability from age-based change is a key challenge here. While the former has been normalized in the template creation process at each age point, variability will persist across age points since the set of subjects across the age points are likely to be mutually exclusive in a cross-sectional setting. Therefore, there is a need to isolate those deformations that arise due to natural aging, and importantly, discard those deformations that come from inter-subject or non-aging variations. One key hypothesis we make is that the inter-subject variability across templates will cause large deformations, and the deformations due to aging are small smooth deformations^20,23,31,32^. Our approach, therefore, identifies a median age point and uses 2-steps: computing pairwise SVFs between given templates which rejects large deformations due to non-aging factor, as SVF (Stationary Velocity Field) based registrations captures only smooth deformations. Next, performing a composition step to filter out residual temporally inconsistent non-aging deformations and derive a single SVF from the median point in the forward and backward directions. The extracted single SVF models a monotonic deformation. A component to capture the rate of aging is introduced in the model to represent the non-uniform nature of the natural aging process. The computed aging deformation is then transported to the global template space to define the final normative aging model. A schematic representation of the proposed aging model in shown in Fig. 1. The construction process is described in detail next.

### Computing the global structural template (*G*)

Given a template sequence $T_i$; $i=1, 2, \dots , N$ representing healthy adults in an age range, in order to capture the aging process, the age range of interest should ideally be sampled adequately, i.e. *N* should be large enough for the sequence to represent normative aging well. We will assume that every template $T_i$ has been constructed over sufficient number of scans of subjects whose age is in the $i{\text {th}}$ interval. As each of the templates can be derived from different sets of images/subjects, the template space defined for each set need not be the same and thus the aging path will be different for each $T_i$. Finding the common aging path from the unaligned $T_1, T_{2},\ldots ,T_{N}$ is the challenge here. The final aging model is therefore defined on a global average template *G* representing all the $T_i$s in the diffeomorphic space ${\mathcal {G}}$. The template *G* is computed using a non-rigid group-wise registration method called SyNG^33^ which iteratively minimizes the average distance from *G* to each of the $T_i$s in the space ${\mathcal {G}}$. The distance incorporates both the non-aging (cross-sectional) and aging deformations and hence *G* normalizes over both these deformations. Considering such a global average as the structural template element in the proposed model avoids biases toward any $T_i$. All the $T_i$s are aligned to *G* using an affine transformation before developing the model. This simplifies the model as an affine alignment can be done accurately from one template to another template.

### Computing the aging deformation

The proposed model does not follow an optimization strategy to find the aging process in global space. It utilises the properties of the aging process in an adult brain, as discussed earlier. Aging deformation across the nearby time/age points is expected to be smooth and small as aging is a slow process. We choose SVF based registration because they are known to extract smoother and smaller deformations compared to Large deformation diffeomorphic metric mapping (LDDMM)^34^ or Free Form Deformation (FFD)^35^ registration models. In SVF based registration methods, the deformation $\phi$ across images is modelled by an SVF *v*, where the group exponential map of *v* gives $\phi$, i.e., $\phi =\exp (v)$.

We define *G* to be the global average representation of all $T_i$s. Since a pairwise mapping between *G* and a template $T_i$ captures both aging and non-aging deformations it cannot be used directly to extract the aging deformation from the $T_i$s. We therefore find a template among $T_i$s that is closest (i.e. smallest deformation) to *G* and use that as a reference to compute the aging deformation. This reference is denoted as $T_M$. If $\{v_i\}$ are the SVFs that map each $T_i$ to *G* then the reference template $T_M$ is found using the norm of the SVF as the norm is a measure of the distance between the templates. If *A* is a vector field and $a_{i,j}$ is an element vector then the norm of *A* denoted as $\left\| A \right\| =\sqrt{\sum _{i}\sum _{j}|a_{i,j}|^2}$. The desired $T_M$ is the template corresponding to the smallest norm $\Vert v_i\Vert$. In other words,

1 $$\begin{aligned} T_M = T_i \text{ where } i \text{ is } \text{ such } \text{ that } \Vert v_i\Vert = \text{ min }\{ \Vert v_k\Vert , 1\le k\le N\}. \end{aligned}$$

#### Extracting deformations which are consistent over the entire age range from the smooth pairwise deformations

In the proposed model, the aging deformation is considered as a consistent temporal relationship across $T_i$s for all *i*, with $T_M$ being considered as the reference template. This temporally consistent aging deformation is derived from the deformations between consecutive template pairs in the forward (*f*) and backward (*b*) directions which are defined with respect to $T_M$. The SVF between the first pair of templates in the forward direction, namely, $(T_M,T_{M+1})$ is denoted as $v_{1_f}$ and the SVf between the next pair $(T_{M+1},T_{M+2})$ is denoted as $v_{2_f}$ and so on. To generalise, $v_{j_f}$ represents the forward deformations between $(T_{M+(j-1)},T_{M+j})$ for all $j=1,2,\dots ,(N-M)$ and $v_{j_b}$ represents the backward deformations between $(T_{M-(j-1)},T_{M-j})$ for all $j=1,2,\dots , (M-1)$ . These SVFs reject all large non-aging variations across age points.

Any smooth non-aging deformations that remains in the above step is generally random across pairs. They can be rejected and temporally consistent aging deformation across templates retained by composing the forward/backward pairwise deformations.

Specifically, we compose the deformations sequentially using the Baker–Campbell–Hausdorff (BCH) formulation^36^ given in Eq. (3) below. Since the BCH formula in Eq. (3) involves the sum of vector fields and its Lie derivatives, the composition has an averaging effect serving to smooth out the random deformations. This allows compositions of group exponentials to be expressed as a single SVF, which models a consistent behaviour across the entire age range in spatial neighbourhoods. Let the velocity vector field obtained as a result of repeated application of BCH formula on the forward (backward) deformations be denoted as $\mathbf {v_{j_f}}$ ($\mathbf {v_{j_b}}$). The vector field $\mathbf {v_{j_f}} \forall j=1,2...(N-M)$ defines the single SVF parameterization of the forward deformation from $T_M$ to $T_{(M+j_f)}$ and $\mathbf {v_{j_b}} \forall j=1,2...(M-1)$ defines the same for the backward deformation from $T_M$ to $T_{(M-j_b)}$. These velocity fields are computed from Eq. (2) with an initialization of $\mathbf {v_{1_f}}=v_{1_f}$ and $\mathbf {v_{1_b}}=v_{1_b}$.

2 $$\begin{aligned} \mathbf {v_{j_f}}= & {} \text{ BCH }(\text{ BCH }(\cdots (\text{ BCH }(\mathbf {v_{1_f}},v_{2_f}),v_{3_f}),...),v_{j_f}), \quad \text {for}~j=2...(N-M),\nonumber \\ \mathbf {v_{j_b}}= & {} \text{ BCH }(\text{ BCH }(\cdots (\text{ BCH }(\mathbf {v_{1_b}},v_{2_b}),v_{3_b}),...),v_{j_b}) , \quad \text {for}~ j=2...(M-1). \end{aligned}$$

The BCH formula for a pair of forward deformations is given in Eq. (3). Backward deformations can be computed in a similar manner.

3 $$\begin{aligned} \text{ BCH }(\mathbf {v_{(j-1)_f}},v_{j_f})= & {} \log (\exp (\mathbf {v_{(j-1)_f}})\exp (v_{j_f}))\nonumber \\= & {} \mathbf {v_{(j-1)_f}}+v_{(j_f )}+\frac{1}{2}([\mathbf {v_{(j-1)_f}},v_{(j_f )}])\nonumber \\&+\frac{1}{12}([\mathbf {v_{(j-1)_f}},[\mathbf {v_{(j-1)_f}},v_{(j_f )}]] +[{v_{j_f}},[{v_{(j_f)}},\mathbf {v_{(j-1)_f}}]])+\cdots =\mathbf {v_{j_f}}. \end{aligned}$$

Here, $[\cdot ,\cdot ]$ denotes the Lie bracket of two vector fields.

It should be noted that since the BCH approximation is valid only for small deformations, in practice, $v_{j_f}$ is divided into *n* smaller deformations such that $\frac{v_{j_f }}{n}< 0.5 \times$ voxel dimension, and these smaller deformations are composed iteratively with $\mathbf {v_{(j_f-1)}}$ to compute $\mathbf {v_{j_f}}$. In the proposed method, the extracted deformation is constrained to be spatially smooth due to the log-Euclidean framework and temporally smooth since the composing step captures only the temporally consistent trends from the sequential data. For simplicity, the forward aging deformation from $T_M$ to $T_N$, $\phi _f=\exp (\mathbf {v_{(N-M)}}t)$ is denoted as $\exp (\mathbf {v_f}t)$ and the backward aging deformation from $T_M$ to $T_1$, $\phi _b=\exp (\mathbf {v_{(M-1)}}t)$ is denoted as $\exp (\mathbf {v_b}t)$.

#### Imposing non-uniform temporal variations on aging deformation

The computed initial-value based aging deformations $\phi _f$ and $\phi _b$ vary uniformly with time which is not consistent with the natural aging trends. For example, tissue degradation is rapid in the elderly age range^23^. Hence, a temporal dependency is introduced in $\phi _f$ and $\phi _b$ to accommodate any non-uniform changes in natural aging. This step is explained next.

**Figure 2:**
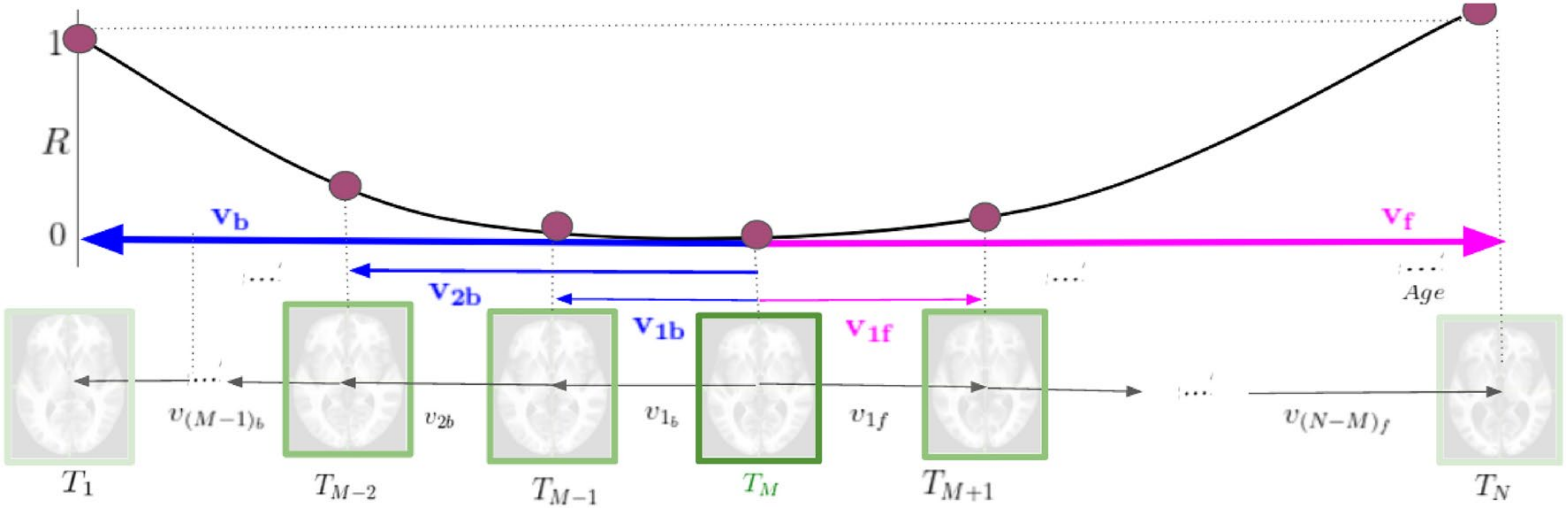
Illustration of the proposed aging deformation computation.

We propose a quantification for the aging deformation (denoted as *R*) at each time point in Eq. (4). This is defined in terms of the distance between $T_i$ and $T_{M}$ as in Eq. (4). A norm helps to quantify the magnitude of the given vector field. Hence, $\left\| \mathbf { v_i} \right\|$ helps to quantify the aging deformation modelled by an SVF $v_i$. With this, let us define

4 $$\begin{aligned} R(j_f)=\frac{\left\| \mathbf { v_{j_f}} \right\| }{\left\| \mathbf {v_f}\right\| } \quad \text {for}~ j=1...(N-M), \qquad R(j_b)=\frac{\left\| \mathbf { v_{j_b}} \right\| }{\left\| \mathbf {v_b }\right\| } \quad \text {for}~ j=1...(M-1). \end{aligned}$$

*R* is a discrete sequence, whereas a continuous aging trend is of interest. This can be achieved by fitting a curve (such as a cubic spline) to *R*(*i*). The resulting function $\gamma (t)$; $t=[t_1,t_N]$, quantifies the aging deformation at a particular time point with respect to $T_M$. As this deformation increases in both directions with time, the curve will, in general, have a bilateral increasing trend about the age point corresponding to $T_M$. The aging deformation computation is illustrated in Fig. 2.

### Transferring the deformations to the global template space

Finally, the deformations captured using Eq. (3) are mapped to the global template space using the mapping from $T_M$ to *G*, i.e., $\exp (v_M)$. The captured deformations on the manifold ${\mathcal {G}}$ are parameterized by SVF. In order to transfer the aging deformations to the global template space we use an existing algorithm^37^ for parallel transport which is explained next.

**Figure 3:**
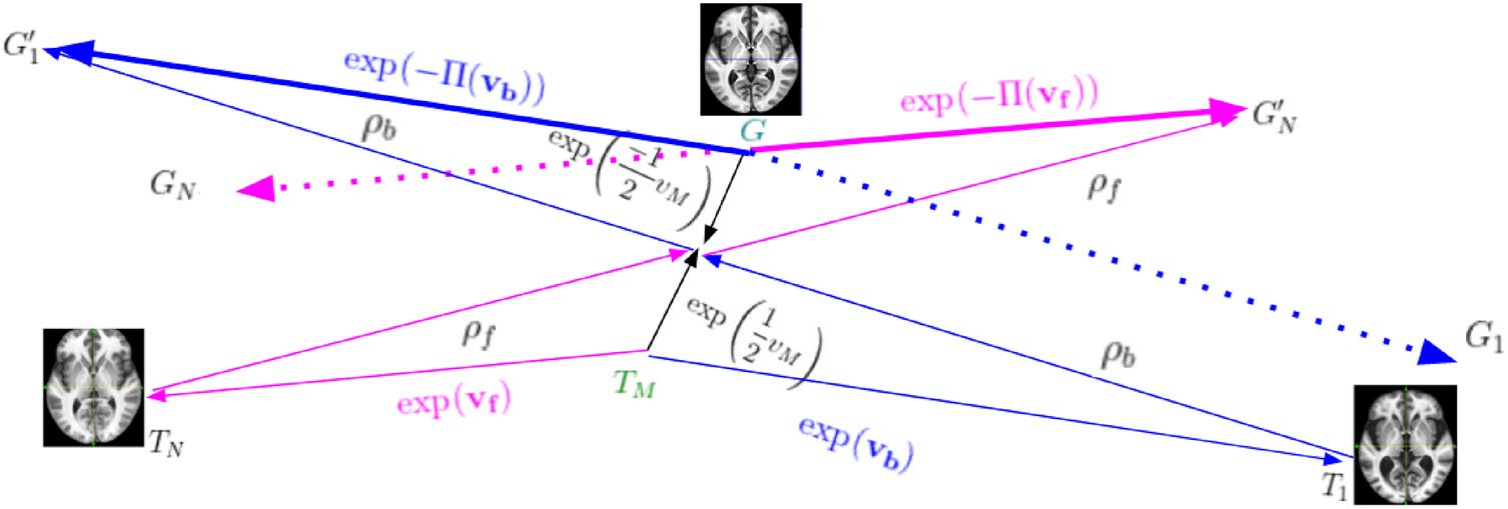
$T_M$ is mapped to *G* using $\exp (v_M)$ and the path is used to transport $\exp (\mathbf {v_f})$ and $\exp (\mathbf {v_b})$ to the global template space.


Let the global template space images corresponding to $T_i$s be $G_i$s. The deformations to be transported are parameterized by SVFs $\mathbf {v_f}$ and $\mathbf {v_b}$. A schematic of the deformation mapping scheme is shown in Fig. 3. In Fig. 3, $G_1'= G \circ \exp (-\Pi (\mathbf {v_b}))$ and $G_{N}'= G \circ \exp (-\Pi (\mathbf {v_f}))$. Thus, the inverse of the mappings from *G* to $G_1'$ and $G_{N}'$ i.e., $\exp (\Pi (\mathbf {v_b}))$ and $(\exp (\Pi (\mathbf {v_f}))$ gives $\phi _b$ and $\phi _f$ respectively. Here $\rho _b=\exp \left( \frac{v_M}{2} \right) \circ \exp (-\mathbf {v_b})$ and $\exp (\Pi (\mathbf {v_b}))=\exp \left( \frac{v_M}{2} \right) \circ \rho _b^{-1}$. Therefore,

5 $$\begin{aligned} \exp (\Pi (\mathbf {v_b}))=\exp \left( \frac{v_M}{2} \right) \circ \exp (\mathbf {v_b})\exp \left( \frac{-v_M}{2} \right) , \end{aligned}$$

and similarly,

6 $$\begin{aligned} \exp ( \Pi (\mathbf {v_f}))=\exp \left( \frac{v_M}{2} \right) \circ \exp (\mathbf {v_f})\exp \left( \frac{-v_M}{2} \right) . \end{aligned}$$

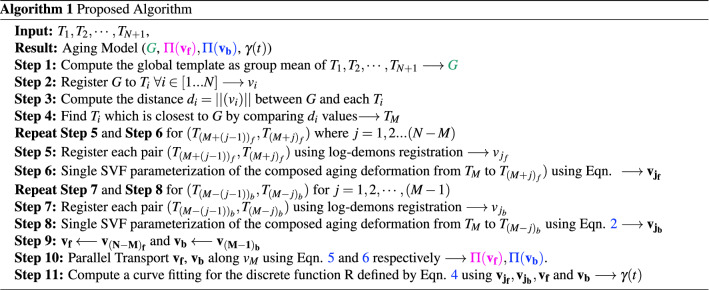


### The aging model

The proposed aging model has three components: *G*, $\gamma (t)$ and the SVF parameterization of the transported forward and backward deformations $\Pi (\mathbf {v_f}), \Pi (\mathbf {v_b})$ respectively. An age-specific template at any time point *t* can be computed using the following formula:

7 $$\begin{aligned} T(t)={\left\{ \begin{array}{ll} G \circ \exp (\Pi (\mathbf {v_f})\gamma (t)) &{}\text{ for } t\ge t_M, \\ G \circ \exp (\Pi (\mathbf {v_b})\gamma (t)) &{}\text{ for } t\le t_M. \end{array}\right. } \end{aligned}$$

The aging model implementation is publicly available at http://doi.org/10.17632/nw983x225c.1.

## Results

Several experiments were conducted to assess/validate the proposed aging model. All experiments, barring the one with simulated data, were done on 3D data though only 2D central slices from the results are shown for visual comparison. The proposed aging model is affine invariant, and, therefore, results were also aligned using affine transformation prior to comparison. The proposed method to create an aging model was implemented using two cross-sectional template datasets covering a wide age range: (i) Brain Imaging of Normal Subjects (BRAINS)^27^ with 7 templates of subjects aged 25–93 years and (ii) Neurodevelopmental MRI Database (Neurodev)^24^ with 14 templates of subjects aged 20–89 years. T1 scans were used in both datasets. More information regarding the subject scans can be found in Refs.^24,27^. The dataset provides information on the age interval and number of scans of subjects were used to create each template. In BRAINS, the sampling of the age range is not uniform, particularly at the upper age level, and the number of scans used for template data creation is less relative to Neurodev. The spacing between template data is shorter (5 years) and uniform in Neurodev.

### Aging model

**Figure 4:**
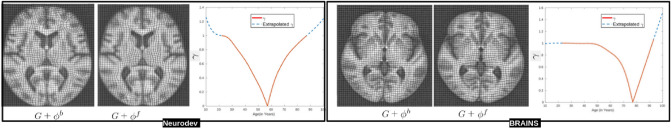
The aging model computed with Neurodev and BRAINS datasets. Sample forward and backward deformations are shown along with the function $\gamma$.

Recall that the proposed aging model has two elements, namely, the structural template *G* and the aging deformation. The aging deformation has three components: the forward aging deformation $\phi _f$ parameterized by $\mathbf {v_f}$, $\phi _b$ parameterized by $\mathbf {v_b}$ and the $\gamma$ function. The proposed model developed with Neurodev and BRAINS datasets are shown in Fig. 4. Understanding aging trend from $\gamma (t)$ plot is not feasible as it represents the degree of deformation with respect to *G*, rather than any of the endpoint templates. It however does indicate the age point that corresponds to the reference template $T_M$; which is 57 years for Neurodev and 77 years for BRAINS.

### Representation quality analysis

Age-specific templates were generated with the proposed aging model using Eq. (7), and were used for visual comparison to assess the quality of representation. Comparisons are done with natural aging trends, existing spatio-temporal atlas and the supplied templates used for model creation.

#### Compatibility with natural aging

**Figure 5:**
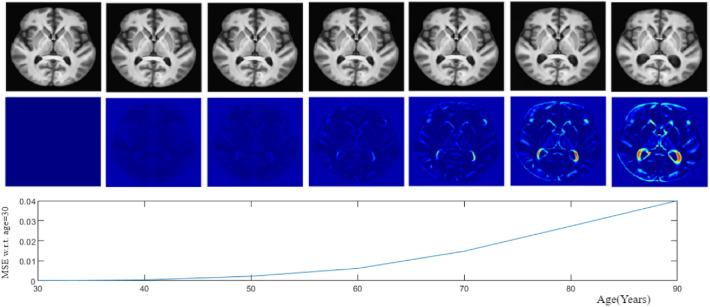
The proposed aging model at different time points(first row) along with the difference image with respect to the initial time point (second row).

Templates at increasing age points were generated with the proposed aging model to study the structural change with aging. The BRAINS dataset^27^ was chosen to do this experiment as it covers a longer span at the elderly age end since the human brain aging literature^23,31,32^ indicates that a mature brain undergoes minimal cognitive and structural changes up to the age of $\approx \,50$ and more for the elderly, i.e. $\approx 60+$. This trend was verified by computing the intensity difference between the current template and the first (at age 30) template. This difference essentially is due to age-induced structural change. Figure 5 shows the generated sequential templates (first row) and difference between the sequential templates and the first template (second row). The difference images facilitate understanding the structural changes with aging. The difference appears to be very low for the first few decades relative to the last few decades where changes like ventricular expansion occurs. This trend is consistent with the existing information about natural healthy aging.

#### Growth trend across aging models

**Figure 6:**
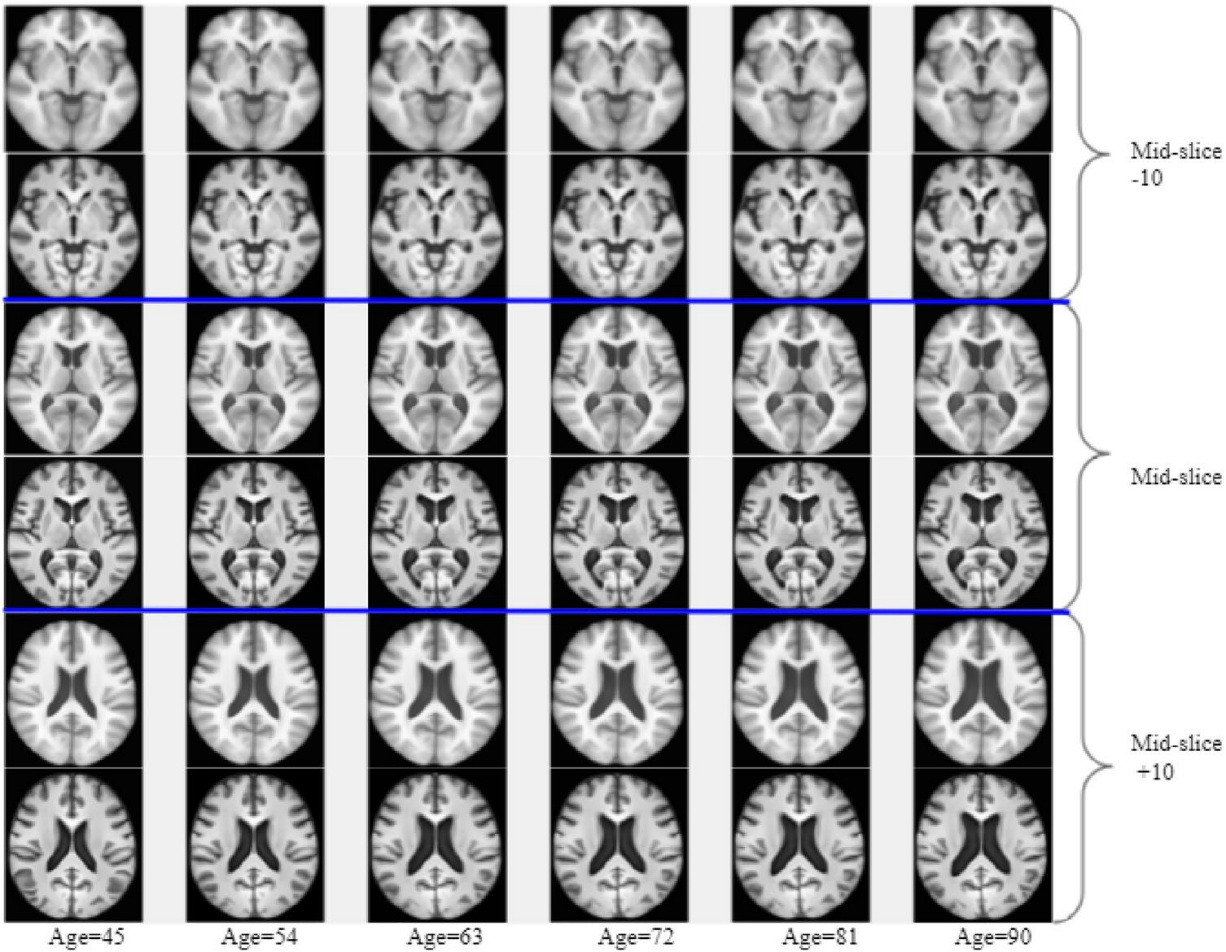
Correctness of aging trends captured in the model: Publicly available spatio-temporal images^14^ (row 1, 3, 5) compared with images generated at same time points with proposed aging model using Neurodev data at different time points (row 2, 4, 6) Each highlighted row pairs compare same slices as specified in the figure.

Huizinga et al. in^14^ proposed a cross-sectional spatio-temporal reference model for representing aging. This model does not ensure a diffeomorphic aging deformation and the template space representation of a subject image needs a computationally intensive group-wise registration with a training set to generate the model. In contrast, our model requires only one pairwise registration from a subject to the corresponding age-specific template, derived from the model. The aging trends observed in the templates derived from our model were compared with those derived using^14^; the latter templates are available in http://www.agingbrain.nl/ for the age range of 45–92 years. Templates at the same age points were generated with the proposed method using the Neurodev dataset. Figure 6, shows sample 2D slices of templates from Ref.^14^ in odd numbered rows, along with the ones derived with the proposed model (from the Neurodev dataset) in even numbered rows, for comparison. The comparison at image-level comparison is not meaningful as the templates are generated from different data-sets. However, one can observe growth trends. The structural similarity across rows in a column appear to have similar trends across age indicating growth trend to be consistent.

#### Aging model assessment

**Figure 7:**
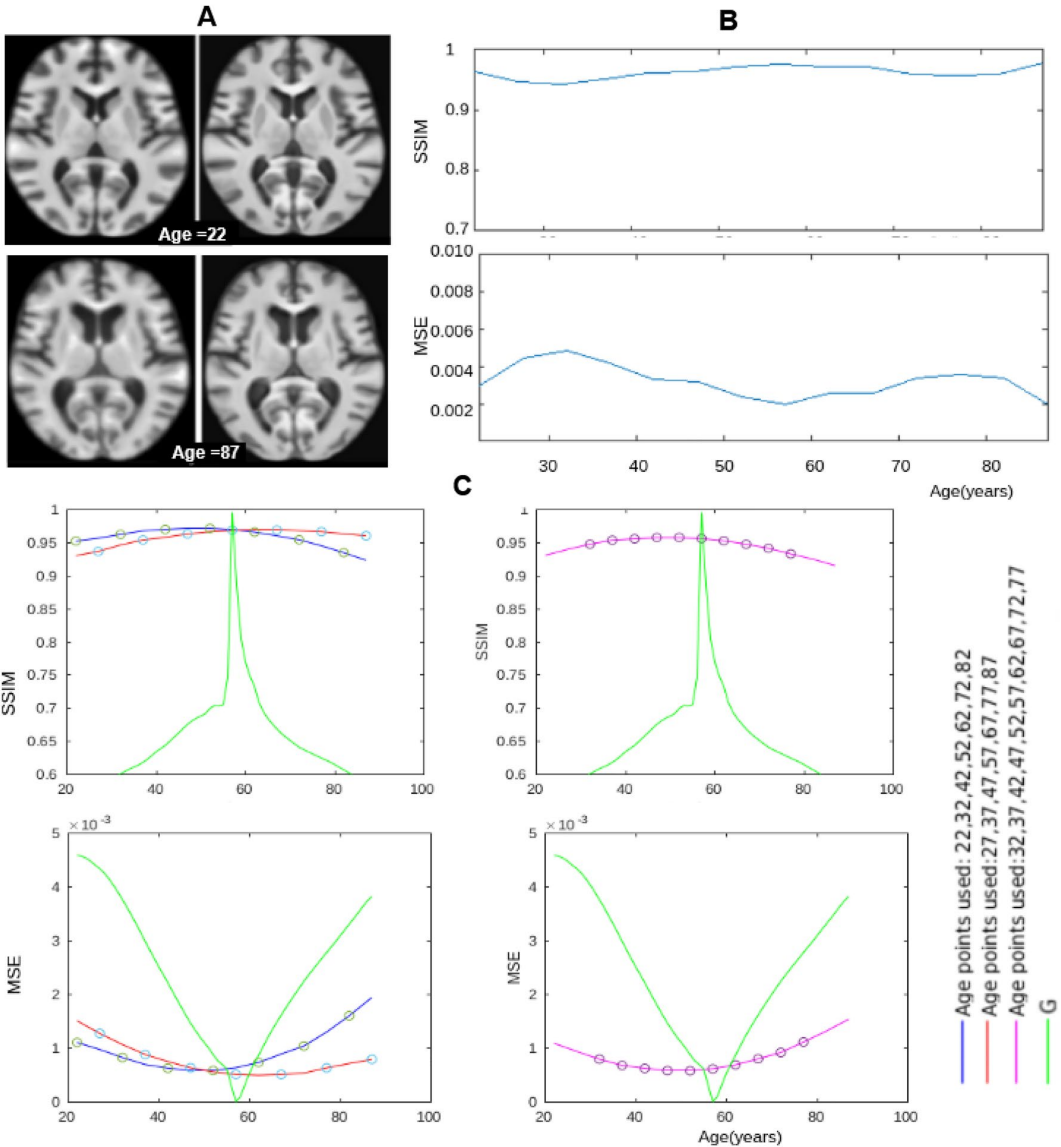
(**A**) Qualitative assessment of templates (first image in each pair) given in the Neurodev dataset against those generated by the proposed aging model (second image in each pair). Only the templates for the first and last time points are shown. (**B**) Quantitative assessment with SSIM (top) and MSE (bottom) values between given templates and generated templates. (**C**) Interpolation (left) and extrapolation (right) assessment with SSIM (top) and MSE (bottom).

The generated templates with our model were visually compared with the templates given in the Neurodev dataset to understand how well the model represented these templates. The templates for the first and last time points in our aging model have undergone maximum deformation compared to those at other age points. Hence, such a visual comparison is of interest. The given templates along with our generated templates are shown in Fig. 7. The first and last time points for Neurodev are shown as the first image in each pair, while the corresponding templates generated by the proposed model are shown as the second image in each pair. The derived templates are seen to be visually quite similar to the templates from the two datasets. This similarity was also quantitatively studied using the structural similarity index (SSIM) and mean square error (MSE). These are reported in Fig. 7B. The high SSIM and low MSE between the templates derived with the proposed model and the given templates show the goodness of fit of the proposed model.

We also assessed the proposed model’s ability to generalise via interpolation/extrapolation experiments. A baseline model was developed with all data (14 templates with sampling interval = 5 years) in Neurodev and compared with the models developed with fewer templates and age range. Two aging models were constructed with 7 templates by skipping alternate templates (sampling interval = 10 years). There are two options to select 7 points from the 14 points in the set, either by skipping odd index points (27,37, ..., 87 years) or by skipping even index points (22, 32, ..., 82 years). In each case, one extreme point (22/87 years) is missed and the range of data is changed. From the SSIM and MSE plots in Fig. 7C, it can be noted that under-sampling and missing data in critical age points leads to a degradation in the performance of the aging model though this is moderate (less than 8$\%$ for SSIM and a maximum MSE of 0.002). The extrapolation capability of the proposed model was also studied by constructing an aging model using templates (sampling interval = 5 years) with a reduced age range, namely 32 to 77 years. This model, therefore, has missing information at both young (22, 27 years) and elderly age (82, 87 years). The SSIM plot shows a dip at the younger and older age points where data is being extrapolated. The error however remains moderately low (less than 9% for SSIM and maximum MSE of 0.0016). In summary, reducing the sampling rate generally leads to a moderate degradation in the overall performance relative to the baseline. Missing data at older age points leads to larger degradation at the elderly age points relative to missing data at a younger age.

### Aging model validation

Model validation was done by analysing the ability of the model to capture natural deformations and the similarity of model-generated age-specific templates to a set of subject images of the same age. Since our model was derived for a cross-sectional setting, we also studied its performance in a longitudinal data setting as it is of interest.

#### Topology preservation

**Figure 8:**
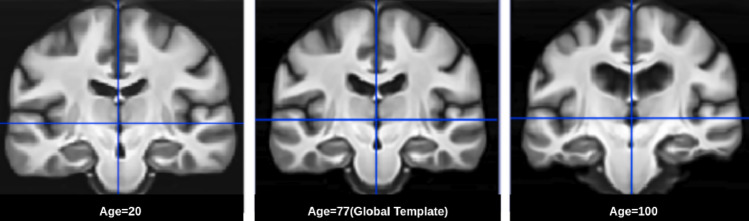
The central coronal slices of extrapolated age-templates are shown along with the global template image.

Since diffeomorphic deformations best fit *natural* deformations, we considered aging related deformation also as a diffeomorphism. Accordingly, our model is defined on a manifold ${\mathcal {G}}$ of diffeomorphisms. It is of interest to verify if an extrapolation of the model generates deformations in ${\mathcal {G}}$ itself. This was done by extrapolating the aging trend and deriving templates in both younger and older ages. Neurodev data which covers that age range of 22–87 (reference template age point, M=77 years) was used for this experiment. The templates from extrapolation in both directions were generated for this experiment with Eq. (7). Two templates, namely at age 20 and age 100, generated with the proposed model are shown in Fig. 8 along with the global template. These are the results of extrapolation from the data given in the BRAINS dataset^27^. The topology appears to be preserved even when the aging model is extrapolated in both directions implying that the extrapolated deformations also belong to ${\mathcal {G}}$. It can also been that while global similarity (in structure) exists across age, local deformations persist. For instance, the ventricle is much smaller at age 20 and enlarges with age, consistent with the expected aging trend. The Jacobians of the forward and backward deformations were verified to be positive valued.

#### Validation with segmentation

A localised assessment by segmenting a few structures is of interest in many situations. This is generally done using a label transfer process wherein a subject image is aligned to a labeled template and the label is transferred. An alignment process that requires smaller deformations indicates that the template is structurally very close to the subject image. This will lead to better segmentation. With our age model, this involves only a single registration step as shown in Fig. 1 and hence potentially involves the least deformation. This is in contrast to the steps required when using the model proposed in Ref.^29^ which requires two registration steps: one to transfer the labels from the global template to the template data that is closest to the given subject age, and a second to transfer the template labels to the subject image. Each of these registration steps can contribute to error in labeling in addition to the structural dissimilarity of template and subject image. An experiment was done to quantitatively compare the accuracy of labeling using the proposed method and with Ref.^29^. From MICCAI 2012 dataset, Ref.^38^ 35 subject images in 18-90 age range along with the ground truth labels were used to perform the comparison. The templates corresponding to the subject ages outside the range (22–87) years, were constructed by extrapolating the proposed aging model. The accuracy of label transfer from a template is highly influenced by the registration method and the global template labels being used. For a fair comparison, both models were developed with Neurodev^24^ templates and the labeled *G* was taken to be identical. Both methods used DRAMMS-based registrations^39^ with identical parameters for label transfer steps. The Dice score was used for assessing the segmentation accuracy.

**Figure 9:**
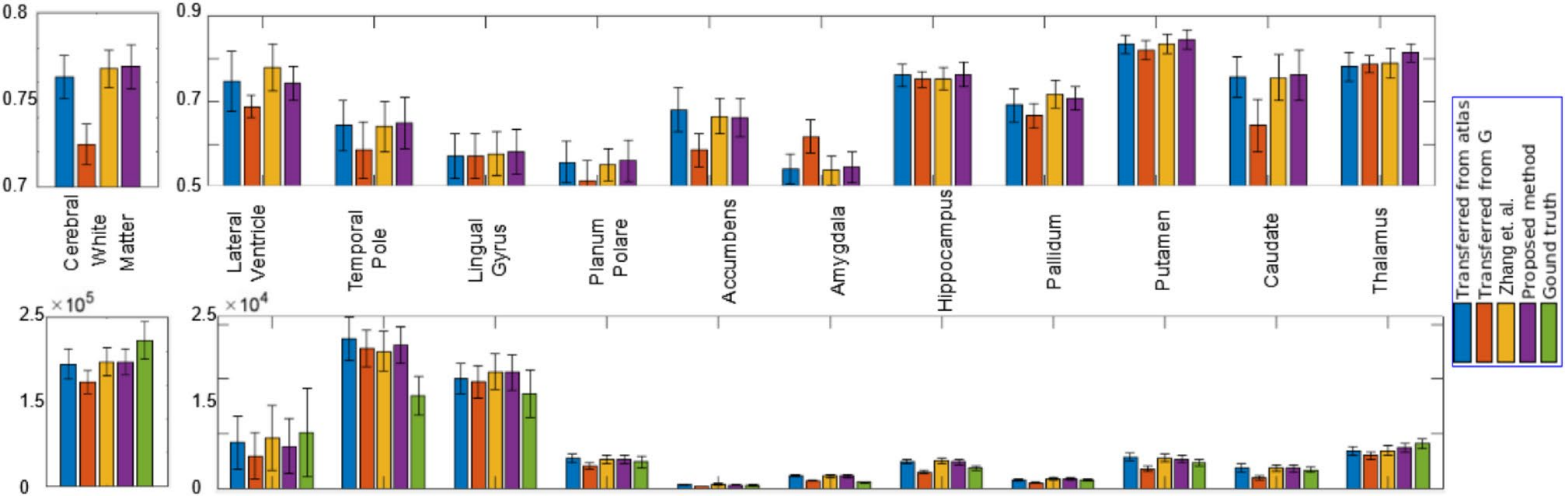
Segmentation performance comparison of the proposed method with Zhang et al.^29^. Baselines considered for this experiment are segmentation results via direct transfer from G and direct transfer from Harvard–Oxford atlas^40^ to the subject images. The average Dice (first row) and volume (second row) are plotted for 12 structure pairs.

Figure 9 shows the Dice scores and volume comparison for cerebral white matter and 11 combined pairs of brain structures for our model and Ref.^29^. The global template *G* was labelled using the Harvard–Oxford atlas^40^ and then transferred to the templates derived with the proposed method and Zhang et al.^29^ as the starting point. It should be noted that in Ref.^29^, a direct manual labelling of *G* was used. Results of direct label transfer from the Harvard–Oxford atlas and *G* are also included to serve as baseline. The difference in segmentation performance between Zhang et al.^29^, labels transferred from atlas method and the proposed method are not statistically significant for almost all structures.

**Figure 10:**
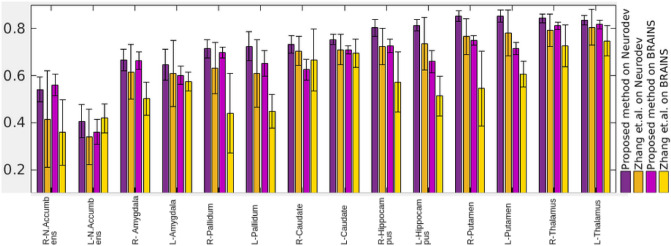
A comparison between performance of the proposed method and Zhang et al.^29^ with Neurodev^24^ and BRAINS^27^ datasets. Dice scores are plotted for various structures.

This experiment was repeated by replacing the Harvard–Oxford atlas^40^ with IBA100 atlas^41^ for both Neurodev^24^ and BRAINS^27^ datasets. The Dice scores are computed with respect to the ground truth available in IBA100 atlas^41^ for 14 sub-cortical structures and reported in Fig. 10, where the difference in Dice values is statistically significant for all structures except L-Amygdala. The proposed method’s segmentation performance is superior on both datasets for most of the structures. A performance reduction is observed for both the methods with the BRAINS dataset compared to Neurodev. This can be due to the fact that Neurodev is densely sampled compared to BRAINS. For Zhang et al.^29^, the average performance reduction with BRAINS is almost twice that of the proposed method. This indicates the superior robustness of the proposed aging model to changes in the sampling of the age range.

#### Validation with simulated longitudinal data

The proposed method was aimed at handling cross-sectional data. In order to understand how the model would handle longitudinal data, an experiment was done using simulations as longitudinal data is unavailable. The Shepp–Logan phantom was used for this purpose and the deformed phantoms were generated as follows. A few locations on the image were chosen and each selected point and its neighborhood were displaced in X and Y directions; the displacement value was sampled from a Gaussian distribution. The degree of deformation is controlled by the parameters of the Gaussian. A set (*S*) of fifty randomly deformed phantoms were taken (to simulate a cohort) and five copies were made. Deformations with increasing degree was applied on these five copies to simulate aging of different subjects. The five sets thus form our longitudinal data. For each of the five sets a template was computed separately using the method suggested in Ref.^42^. The templates were then used as inputs for the proposed model to generate templates at different age points. These were then compared against the deformed versions of the Shepp-Logan phantom (proxy ground truth).

**Figure 11:**
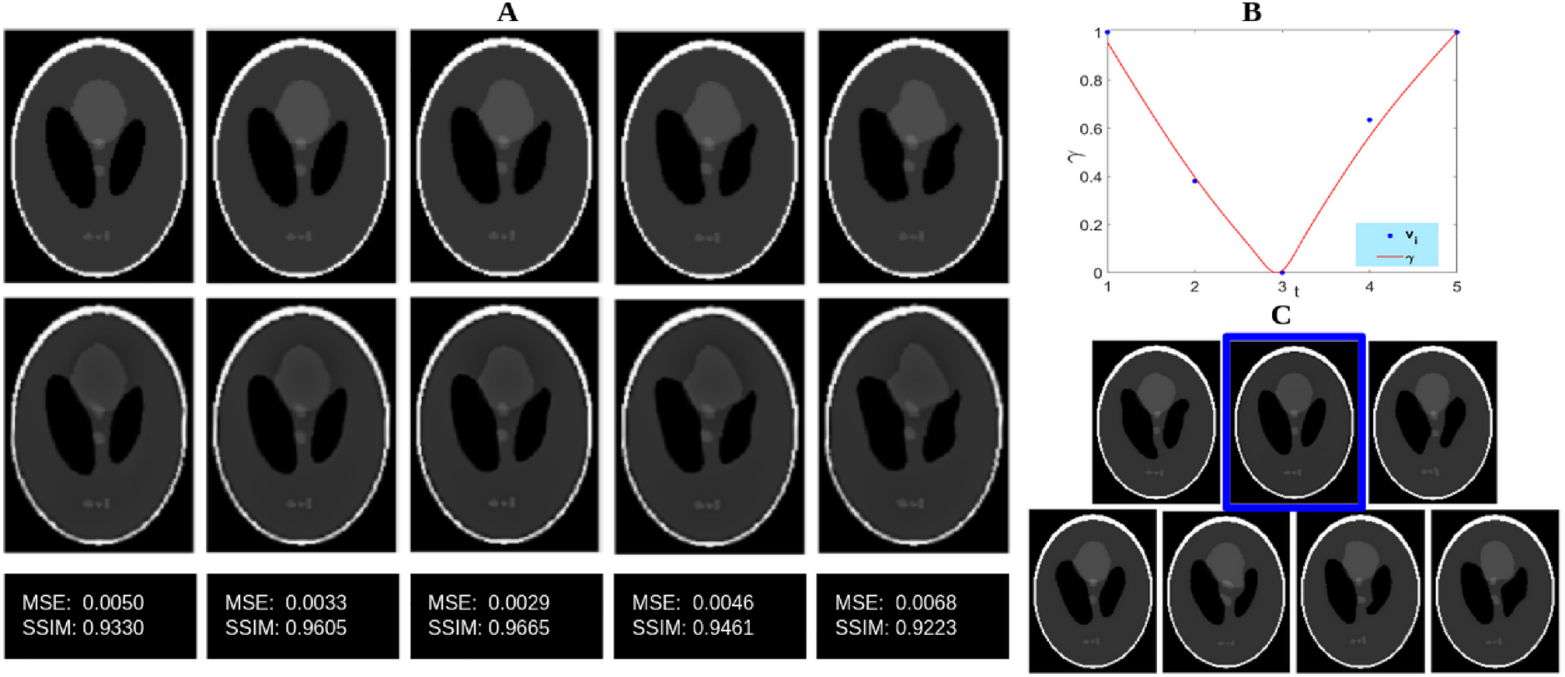
Aging model for a simulated longitudinal dataset. (**A**) First row: Deformed images with known transformation and second row: images generated with the proposed model, for the same time points (as in the first row) and last row: the MSE and SSIM of first and second rows; (**B**) The $\gamma$ curve of our aging model and (**C**) some sample images used in *S*.

Sample images generated by applying the simulated deformations on the Shepp–Logan phantom are shown in the first row of Fig. 11. This forms the ground truth. The template images derived with the proposed model are shown in the second row. The images in the two rows appear to be very similar to each other at the same time points. The template images generated with the proposed model and corresponding $\gamma$ curve is also shown in the same figure. The degree of deformation in the simulated deformation is uniformly increasing with time and hence it can be expected that the $\gamma$ curve will be symmetric with respect to mid-time point. We see that, in Fig. 11, this is indeed true. The proposed model captures the applied deformation without much errors from the simulated longitudinal data. A slight increase in image-level errors is observed with respect to the given data towards the end time points in the model-generated images, which could be due to truncation in Eq. (3).

## Discussion

Cross-sectional images at different age points are easier to acquire than that of the same subject. This motivated us to develop a method to generate a normative aging model for adults using cross-sectional data. The aging model is based on continuous deformation applied to a global average template. Since aging has been shown to result in smooth deformations^20,23,31,32^ the proposed method uses SVF parametrization for pairwise registration between $T_i$s since SVF is limited to capturing only smooth deformations. The composition step extracts the consistent trends from the pairwise SVFs and hence, helps to filter out any smooth, non-aging deformation captured in the pairwise registration step. Thus, the chance of error accumulation from non-aging deformation with the proposed model is quite low. Opting for a two-piece, (forward and backward deformation) strategy also aids in reducing the modelling error and chance of error accumulation. Experimental results show that our aging model can be used to generate templates at any time point $T_i$s in a manner that is consistent with the natural aging trend observed by other studies.

The proposed aging model has a few limitations. Since it was developed for modelling normative aging in the adult brain, modelling cross-sectional data with underlying large or non-monotonically behaving deformations is not possible and will require a redesign of the deformation calculation step. Since the proposed model is data-dependent, the number of scans in each age interval needs to be large enough to generate representative templates. Though the definition of ‘large’ remains open, current studies have typically used 20 or more scans at every interval. Further, while the model reduces the effect of cross-sectional data induced variations in the aging deformation, there is no formal proof as yet that it completely removes the cross-sectional variation. It should also added that the proposed model models only the structural variation with aging whereas cortical thickness, signal intensity variation^43^ etc. have also been used to analyse aging process. Finally, the proposed aging model defines a single average growth path and does not attempt to model the cross-sectional aging variations.

A future refinement of the proposed model can be to attempt modelling aging as a distribution of paths about an average path. This would however require the availability of scans and not templates at different age points. Our current work is directed at developing a public database for this purpose with subject scans at different age points.

The spatio-temporal smoothness and consistency assured in the proposed model make it closer to natural aging. Consequently, the model has the potential to be used for clinical purposes. Currently, population specific aging trends are of interest and this can be generated with the proposed model with minimum effort. The code to generate proposed the aging model has been made publicly available at http://dx.doi.org/10.17632/nw983x225c.1.
